# Placental Pathological Findings and Clinical Outcomes in Triplet Pregnancies Conceived via Oocyte Donation and Non-Oocyte Donation: A Case–Control Study

**DOI:** 10.3390/diagnostics15212681

**Published:** 2025-10-23

**Authors:** Eva Manuela Pena-Burgos, Maria De la Calle, Jose Juan Pozo-Kreilinger, Cecilia García-Díaz, Rita María Regojo-Zapata

**Affiliations:** 1 Pathology Department, Gregorio Marañón General University Hospital, 28007 Madrid, Spain; 2 Obstetrics and Gynecology Department, La Paz University Hospital, 28046 Madrid, Spain; 3 Pathology Department, La Paz University Hospital, 28046 Madrid, Spain; 4 Obstetrics and Gynecology Department, Gregorio Marañón General University Hospital, 28007 Madrid, Spain

**Keywords:** assisted reproductive technology, oocyte donor, triplet pregnancies, triplet placentas, placental evaluation

## Abstract

**Objective**: This study aimed to assess whether oocyte donation in triplet pregnancies is associated with increased risk of placental abnormalities and pregnancy complications compared to triplet pregnancies conceived through assisted reproductive technology (ART) without oocyte donation. **Methods**: This single-center, retrospective, case–control study analyzed triplet pregnancies conceived via ART. The case group included pregnancies resulting from oocyte donation, while the control group comprised triplet pregnancies conceived by ART without oocyte donation. Maternal, obstetric, fetal, and neonatal outcomes were assessed. Gross and histopathological placental findings were evaluated using standardized criteria. Univariate and multivariate statistical analyses were performed. **Results**: A total of 77 triplet pregnancies (231 fetuses) were included: 29 in the oocyte donation group (87 fetuses) and 48 in the non-oocyte donation group (144 fetuses). Multivariate analysis revealed significantly higher rates of pregnancy-induced hypertension (*p* = 0.03), preeclampsia (*p* = 0.03), fetal growth restriction (*p* = 0.04), and fetal death (*p* = 0.01) in the oocyte donation group. Placental evaluation showed a higher frequency of infarcts (*p* = 0.04) and chronic inflammatory lesions—chronic villitis (*p* = 0.02) and chronic deciduitis (*p* = 0.03)—as well as signs of fetal vascular malperfusion, including avascular villi (*p* = 0.02) and stromal–vascular karyorrhexis (*p* = 0.01). Intervillous fibrin deposition was also more common in this group (*p* = 0.02). **Conclusions**: Oocyte donation in triplet pregnancies is associated with increased rates of placental abnormalities and adverse maternal and fetal outcomes when compared with ART without oocyte donation. Placental examination may provide valuable insights into the mechanisms involved. Further research is warranted to clarify the underlying immunological and vascular pathways. **Synopsis**: In our cohort of 77 triplet pregnancies, those conceived via oocyte donation showed significantly higher rates of preeclampsia, fetal growth restriction, and fetal death. Placental examination revealed more chronic villitis, deciduitis, intervillous fibrin, avascular villi, and stromal–vascular karyorrhexis, suggesting immune and vascular dysfunction in oocyte donation pregnancies.

## 1. Introduction

Triplet pregnancies have become increasingly common in recent decades, largely as a consequence of the widespread use of assisted reproductive technology (ART) [1]. In our healthcare region, they represent 0.15% of all pregnancies [2]. These gestations are classified as high-risk [3] and are associated with substantially higher rates of maternal and fetal complications compared with singleton and twin pregnancies [4,5].

Importantly, the incidence of multiple gestations is higher in oocyte donation cycles than in autologous cycles [6], and these pregnancies have been linked to an increased risk of adverse maternal and perinatal outcomes [7,8,9].

Clinical studies have consistently reported that pregnancies achieved via oocyte donation—both singleton [10,11,12,13,14] and twin gestations [15]—present higher rates of preeclampsia and gestational hypertension. These pregnancies also show increased risks of preterm birth and low birthweight [13,16,17]. However, few studies have included triplet pregnancies in their analyses [11], and the specific risks in this population remain poorly defined.

Placental pathology may provide critical insights into the mechanisms underlying these complications. Several studies have described distinct histopathological features in placentas from oocyte donation pregnancies, including chronic villitis, deciduitis, perivillous fibrin deposition, and vascular malperfusion [18,19,20,21,22]. Yet, most of these findings come from singleton or twin pregnancies. Only 22 triplet pregnancies have been included across previous placental studies in the context of oocyte donation [19,21,23].

To our knowledge, no prior study has specifically compared gross and microscopic placental findings between oocyte donation and non-oocyte donation triplet pregnancies. Given the clinical importance of placental function in pregnancy outcomes, this study aimed to determine whether triplet pregnancies conceived through oocyte donation exhibit a higher risk of placental abnormalities and pregnancy complications compared with those conceived through ART without oocyte donation.

## 2. Materials and Methods

### 2.1. Study Design and Eligibility Criteria

This was a retrospective cohort study comparing triplet pregnancies conceived through oocyte donation [in vitro fertilization (IVF) or intracytoplasmic sperm injection (ICSI)] with triplet pregnancies conceived through non-oocyte donation ART (IVF or ICSI), whose placentas were submitted to the Pathology Department for evaluation between January 2000 and April 2024. Clinical data were obtained from the obstetric medical records of triplet pregnancies followed at the Maternal–Fetal Medicine Unit of a tertiary referral hospital for multiple pregnancy management, following approval by the hospital’s Research Ethics Committee (PI-5286 2022.168, approval date: 14 July 2022). The study was conducted in accordance with the Declaration of Helsinki. Patient consent was waived due to the retrospective and observational nature of the study, which involved only anonymized data and did not entail any additional procedures or interventions. The diagnosis of triplet pregnancy, along with the assessment of amnionicity, chorionicity, and gestational age, was carried out by experienced obstetric sonographers during the first and second trimesters. Chorionicity was subsequently confirmed after delivery through pathological examination of the placenta.

### 2.2. ART Procedures and Oocyte Donor Selection

In our healthcare region, oocyte donation is the ART method of choice for women over 40 years of age with diminished ovarian reserve, women over 42 years of age regardless of reserve, and younger women with premature ovarian failure. None of the patients had previously frozen their oocytes. Donated oocytes were selected from a bank of women under 35 years of age, with no history of conditions such as diabetes or hypertension. All oocytes used had been cryopreserved. Preimplantation genetic testing and human leukocyte antigen (HLA) compatibility testing were not routinely performed. Recipients were treated with high-dose estrogen therapy, and no immunomodulatory medications were administered before or during pregnancy. Low-dose aspirin was prescribed prior to embryo transfer in cases from 2012 onward.

### 2.3. Basal Study Variables

Baseline data included chorionicity, maternal age at conception, pre-pregnancy body mass index (BMI), year of conception, preexisting medical conditions associated with subfertility, previous failed pregnancy attempts (including previable intrauterine losses), and parity.

### 2.4. Maternal, Obstetric and Neonatal Outcomes

Maternal complications assessed in this study comprised preeclampsia, pregnancy-induced hypertension, gestational diabetes, intrahepatic cholestasis, hypothyroidism of pregnancy, and iron-deficiency anemia. Fetal complications included fetal growth restriction (FGR), twin-to-twin transfusion syndrome, twin anemia–polycythemia sequence, biometric and amniotic fluid discordance, congenital malformations, and intrauterine death. Additional obstetric variables recorded were threatened preterm labor, premature rupture of membranes, and preterm birth (<34 weeks). Perinatal outcomes included neonatal death, neonatal weight, umbilical cord pH, and 5-min Apgar score.

### 2.5. Gross Placental Evaluation and Gross Variables

Gross placental data were extracted from pathology reports. Variables recorded included placental weight, maximum diameter, and placental thickness (measured at the thickest area). In cases with a monochorionic component, the presence of superficial vascular anastomoses was documented. Additional parameters were the type of umbilical cord insertion (central, paracentral, marginal, or velamentous), cord length, thickness, and coiling pattern. Cord coiling was classified as hypocoiling (<1 coil per 10 cm), normal (1–8 coils per 10 cm), or hypercoiling (>8 coils per 10 cm). The presence of a single umbilical artery, true knots, membranous abnormalities (yellowish or greenish discoloration), hematomas (intraparenchymal, retroplacental, or subchorionic), and placental accreta were also assessed. Placental weight was further categorized into four groups (≤10th percentile, 11th–50th percentile, 51st–89th percentile, and ≥90th percentile) according to reference values established by Pinar et al. [24]. Deep vascular anastomoses were not evaluated.

### 2.6. Pathological Examination and Pathological Variables

All available material from pathology records was reviewed, including hematoxylin and eosin–stained slides from the umbilical cords (one slide per fetus), membranes (one slide per fetus), decidua (one slide per fetus), umbilical cord insertion sites (one slide per fetus), and villous parenchyma from each placental disc or sector, according to chorionicity. From 2000 to 2016, one villous parenchyma slide per fetus was examined; from 2017 to 2024, two slides per fetus were analyzed. Tissue samples were fixed in 10% neutral buffered formalin for 24–48 h, embedded in paraffin, and sectioned at 5 μm. All samples were examined by two experienced pathologists, and final diagnoses were confirmed by consensus. Histopathological findings were categorized according to the Amsterdam Placental Workshop Group Consensus Statement and its updated criteria [25,26]. Diagnoses were classified into three main categories. Inflammatory lesions included acute chorioamnionitis (stage and grade), acute funisitis (stage and grade), chronic villitis of unknown etiology (VUE) (grade), chronic intervillositis, and chronic deciduitis. Vascular lesions encompassed accelerated villous maturation, distal villous hypoplasia, decidual arteriopathy, vascular thrombi, avascular villi (not associated with villitis), stem vessel obliteration, stromal–vascular karyorrhexis, and intramural fibrin deposition. Other findings comprised placental edema (focal or generalized), chorangiosis, chorangioma, dystrophic calcifications, intervillous fibrin deposition, and the presence of nucleated red blood cells beyond 20 weeks of gestation.

All stages of acute chorioamnionitis, acute funisitis, and chronic villitis were pooled for statistical analysis. Only histopathological variables considered evaluable were included in cases with autolysis of placental discs or sectors.

### 2.7. Statistical Analysis

Quantitative variables were summarized as means with standard deviations when normally distributed, or as medians with interquartile ranges when non-normally distributed. Qualitative variables were presented as frequencies and percentages. The Kolmogorov–Smirnov (K–S) test was applied to evaluate the normality of quantitative data. Comparisons of qualitative variables were performed using the Chi-square test or Fisher’s exact test when the expected frequency in any cell was <5. Quantitative variables were compared using Student’s *t*-test for normally distributed data or the Mann–Whitney U test for non-normally distributed data. Associations between categorical variables were expressed as odds ratios (OR) with 95% confidence intervals (CI).

Not all data were available for all patients; cases with missing data were excluded from the corresponding analyses. To identify potential confounders associated with pregnancy complications, a multivariate logistic regression model was constructed (Cox R^2^ = 27.4%) using “oocyte donation vs. non-oocyte donation” as the dependent variable. Independent variables were those that showed a statistically significant difference in univariate analysis. These included: previous failed ART pregnancy attempts, preeclampsia, pregnancy-induced hypertension, intrahepatic cholestasis, fetal growth restriction, fetal death, intraparenchymal infarcts, chronic deciduitis, chronic villitis, chronic intervillositis, vascular thrombi, avascular villi, stem vessel obliteration, stromal–vascular karyorrhexis, intramural fibrin deposits, chorangioma, and intervillous fibrin deposits. All statistical tests were two-tailed, and a *p*-value < 0.05 was considered statistically significant. Statistical analysis was performed using SPSS software, version 25 (SPSS Inc., Chicago, IL, USA).

## 3. Results

**Figure 1:**
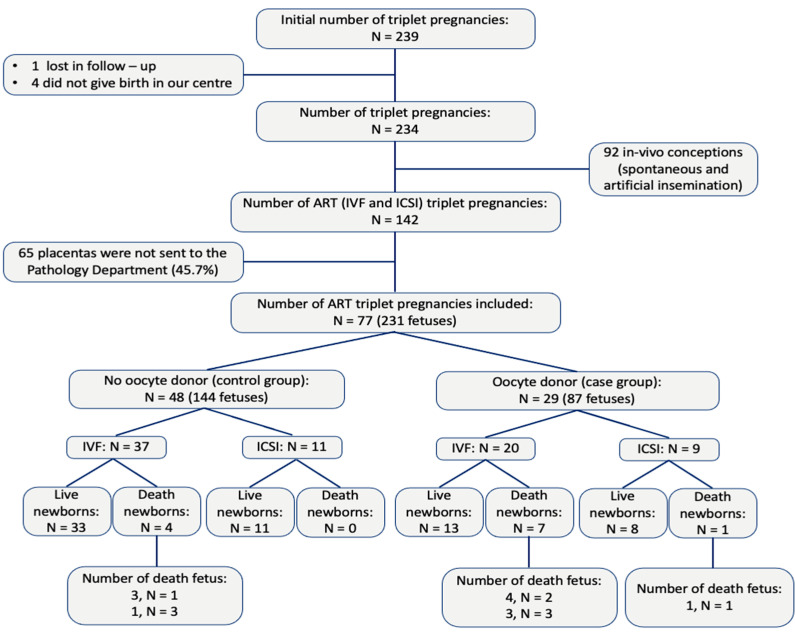
Inclusion and exclusion criteria. Number of placentas analyzed.

A total of 239 women with triplet pregnancies were initially identified; however, five patients were excluded—one due to relocation during prenatal care and four who did not deliver at our hospital. Of the remaining 234 pregnancies, 92 were conceived in vivo (spontaneously or via artificial insemination). Among the 142 ART pregnancies, 65 placentas were not submitted to the Pathology Department. Ultimately, 77 women were included in the study: 48 (62.3%) in the control group (non-oocyte donation) and 29 (37.6%) in the oocyte donation group. Twelve pregnancies experienced intrauterine death, accounting for 24 fetal losses (Figure 1). They occurred between 9 and 27 weeks of gestation, with 20% in the first trimester and 80% in the second trimester. The causes were either spontaneous miscarriage or selective cord occlusion indicated for severe fetal growth restriction or major malformations.

### 3.1. Baseline Characteristics

Chorionicity showed no statistically significant differences between groups. When cases were grouped by the presence of a monochorionic component (MCTA or DCTA), no differences were observed (Table 1). Maternal age was normally distributed (K–S test, *p* = 0.20), with no significant differences between groups (*p* = 0.31). Pre-pregnancy maternal BMI was also normally distributed (K–S test, *p* = 0.20), with no significant group differences (*p* = 0.27). Twenty-eight cases (47.5%) had a history of failed ART pregnancy attempts, which remained significantly more frequent in the oocyte donation group after adjustment (adjusted *p* = 0.03; aOR = 2.34 [1.06–8.76]). There were no significant differences in year of conception (*p* = 0.39) or parity (*p* = 0.06) between groups (Table 1).

### 3.2. Maternal and Fetal Complications

After adjustment (Table 2), preeclampsia (adjusted *p* = 0.03; aOR = 5.02 [2.24–13.42]) and pregnancy-induced hypertension (adjusted *p* = 0.03; aOR = 6.32 [1.76–26.54]) were significantly more frequent in the oocyte donation group. A higher rate of intrahepatic cholestasis lost statistical significance after adjustment (adjusted *p* = 0.12). Fetal growth restriction was significantly more common in the oocyte donation group (adjusted *p* = 0.04; aOR = 3.23 [1.21–6.54]). Among the 231 fetuses, malformations were observed in three (1.4%), including aberrant right subclavian artery, Down syndrome, and Tetralogy of Fallot, with no group differences. Intrauterine death occurred more frequently in the oocyte donation group (adjusted *p* = 0.01; aOR = 7.21 [2.21–16.54]). Fetal losses occurred between 9 and 24 weeks of gestation: 28% in the first trimester and 72% in the second, due either to spontaneous loss or selective cord occlusion. No significant differences were observed for other fetal complications, including threatened preterm labor (*p* = 0.41) and premature rupture of membranes (*p* = 0.56). Preterm birth was assessed in the 73 pregnancies with at least one surviving fetus. Gestational age at delivery was not normally distributed (K–S test, *p* < 0.01), with no significant group differences (*p* = 0.91), including after stratification. Birthweight was not normally distributed (K–S test, *p* < 0.01), and no significant group differences were found (*p* = 0.52). Similarly, no differences were observed after stratification. Umbilical cord pH was not normally distributed (K–S test, *p* < 0.01), with no significant differences (*p* = 0.06). Five-minute Apgar scores also did not differ significantly between groups (*p* = 0.38) (Table 2).

### 3.3. Gross Placental Findings

Placental weight was normally distributed (K–S test, *p* = 0.23), with no significant differences between groups (*p* = 0.45), including after stratification (Table 3). Placental size and thickness were not normally distributed (K–S test, *p* < 0.01 for both), with no significant group differences (*p* = 0.41 and *p* = 0.65, respectively). Superficial vascular anastomoses were assessed in 21 of 40 placentas with a monochorionic component. No differences were found after adjustment (*p* = 0.67). All identified anastomoses were artery-to-artery or vein-to-vein. Among 216 umbilical cords analyzed, no significant differences were observed in insertion type, length (K–S test, *p* < 0.01; *p* = 0.97), or thickness (K–S test, *p* < 0.01; *p* = 0.23). Abnormal coiling patterns were not statistically different between groups (*p* = 0.20 and *p* = 0.23). No differences were found in the frequency of true knots (*p* = 0.63) or single umbilical artery (*p* = 0.88). Membranous alterations (*p* = 0.88) and subchorionic hematomas (*p* = 0.94) also did not differ. No retroplacental hematomas were detected. However, intraparenchymal infarcts or hematomas were significantly more frequent in the oocyte donation group (adjusted *p* = 0.04; aOR = 3.43 [1.65–7.54]).

### 3.4. Histopathological Findings

**Figure 2:**
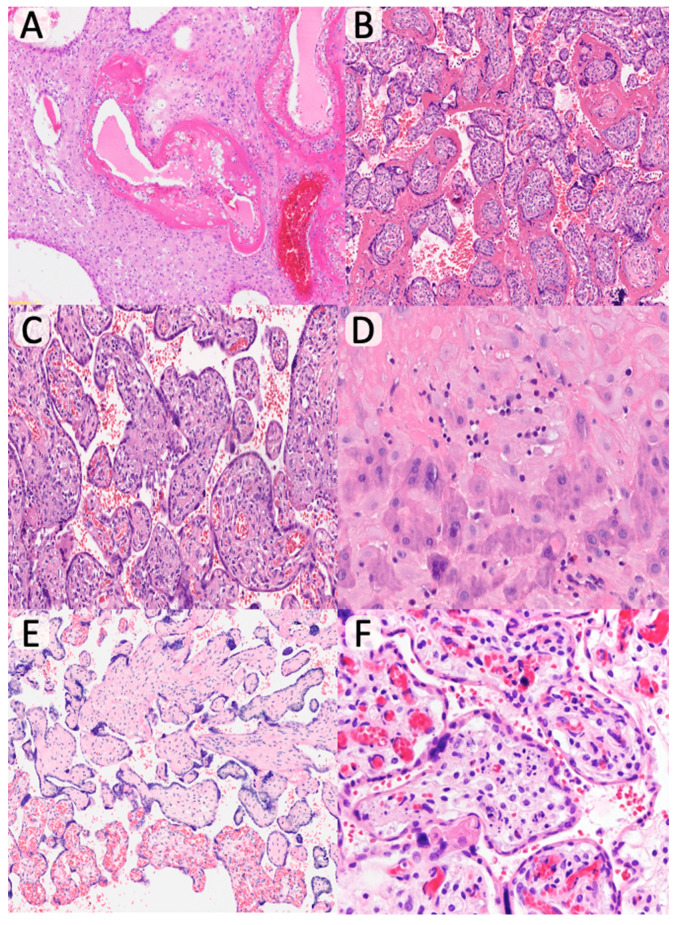
Histopathological findings of oocyte donation pregnancies. (**A**) Decidual vessels with fibrinoid necrosis (H & E, ×200); (**B**) Increased acelular perivillous fibrin deposits (H & E, ×100); (**C**) Chronic lymphoplasmacytic villitis (H & E, ×200); (**D**) Chronic plasma cell deciduitis (H & E, ×400); (**E**) Foci of fibrous and avascular distal villi (H & E, ×100). (**F**) Stromal-vascular karyorrhexis (H & E, ×400).

No differences were found in the acute inflammatory pattern: acute chorioamnionitis (*p* = 0.85) and acute funisitis (*p* = 0.44). However, chronic inflammatory lesions were more frequent in the oocyte donation group: chronic deciduitis (adjusted *p* = 0.03; aOR = 3.24 [1.32–8.78]) and chronic villitis (adjusted *p* = 0.02; aOR = 4.21 [1.56–10.65]) (Table 4, Figure 2). Differences in chronic intervillositis did not remain significant after adjustment (adjusted *p* = 0.06; aOR = 0.45 [0.34–4.34]). Among the villitis cases, 12 were low-grade (8 focal, 4 multifocal), and 3 were high-grade (2 patchy, 1 diffuse). No microbiological agents were identified. Regarding vascular pathology, no significant differences were found in maternal vascular malperfusion markers. However, fetal vascular malperfusion lesions were significantly more frequent in the oocyte donation group. After adjustment, avascular villi (adjusted *p* = 0.02; aOR = 7.82 [2.21–19.09]) and stromal–vascular karyorrhexis (adjusted *p* = 0.01; aOR = 8.65 [3.01–18.76]) remained statistically significant. No significant differences were found in the prevalence of chorangiosis (*p* = 0.36). Although chorangiomas were more frequent in the oocyte donation group, the difference lost significance after adjustment (adjusted *p* = 0.06; aOR = 4.56 [0.96–10.45]); they measured 0.5 to 2 cm in size. Intervillous fibrin deposition was significantly more frequent in the oocyte donation group (adjusted *p* = 0.02; aOR = 2.78 [1.21–6.56]). No differences were observed in the presence of dystrophic calcifications, villous edema, or nucleated red blood cells. No cases of placental accreta were identified.

## 4. Discussion

Our study revealed that oocyte donation (IVF and ICSI) triplet pregnancies presented higher rates of pregnancy-induced hypertension, preeclampsia, fetal growth restriction, and fetal death, as well as more gross intraparenchymal infarcts/hematomas, chronic inflammatory findings (chronic villitis and chronic deciduitis), and fetal vascular malperfusion features (avascular villi and stromal–vascular karyorrhexis) in the placentas.

In this study, 77 triplet placentas (corresponding to 231 fetuses) were analyzed. To the best of our knowledge, this is the first specifically reported cohort focusing on gross and histopathological placental findings related to oocyte donation in triplet pregnancies. Esteves et al. included 16 triplets [21], Gundogan et al. included four [23], and Perni et al. included two [19].

In our series, 74.0% of triplet pregnancies were first pregnancies, consistent with previous studies on triplet outcomes [27,28]. Prior failed ART pregnancy attempts were significantly more common in oocyte donation patients, also in line with the literature [21,23]. No differences were found in the year of conception, and the proportion of oocyte donation cases remained stable over time. An increased number of placentas have been submitted for pathological examination in recent years, as awareness of the importance of placental evaluation in pregnancy outcomes has grown [25,29]. No differences in chorionicity or the presence of a monochorionic component were found. The recent shift toward transferring one or two embryos has resulted in more MCTA and DCTA placentas, while earlier practices of transferring three embryos were associated with higher TCTA rates. A monochorionic component in triplet pregnancies is linked to poorer outcomes, primarily due to vascular anastomoses [30,31,32]. We evaluated superficial vascular anastomoses and found no differences between groups. These anastomoses are believed to offer protection against TTTS [33]. However, we did not assess deep arterio-venous anastomoses, which are implicated in TTTS development, as their evaluation is not routinely performed [34].

The median maternal age was 35.5 years and was slightly higher in the non-oocyte donation group, although the difference was not statistically significant. In contrast, a recent study found higher maternal age in oocyte donation pregnancies across singletons, twins, and triplets [35]. This contradiction may be due to the fact that non-donor patients in our study had cryopreserved embryos at younger ages. Advanced maternal age has been associated with maternal vascular lesions [36] and fetal vascular malperfusion [37]. The median pre-pregnancy BMI was 24.2 kg/m^2^ and slightly higher in the oocyte donation group, although this association has not been consistently reported [35]. Obesity is a known risk factor for pregnancy-induced hypertension [38]. In our study, pregnancy-induced hypertension and preeclampsia were more frequent in the oocyte donation group, consistent with prior reports [8,23], although a recent triplet-focused study did not confirm these associations [35]. One study found an increased risk of severe maternal morbidity in twin pregnancies from oocyte donation [9]. Multiple gestation [15] and diminished ovarian reserve [39] are established risk factors for preeclampsia. These maternal complications may explain the higher rates of fetal growth restriction and fetal death in the oocyte donation group, despite the absence of significant maternal vascular malperfusion markers.

Fetal growth restriction and fetal death were more common in the oocyte donation group, although these outcomes have not been consistently reported in the literature [10,11,12]. In our cohort, up to 20% of oocyte donation pregnancies resulted in fetal death. Triplet pregnancies may inherently carry a higher risk of fetal loss. The increased frequency of maternal vascular complications and fetal malperfusion in the oocyte donation group may contribute to this finding. No significant differences in preterm birth were observed, consistent with previous studies [18,19]. Although oocyte donation has been associated with preterm birth in singleton and twin pregnancies [35], this was not seen in our cohort. VUE, maternal vascular malperfusion, and amniotic fluid infection have been linked to preterm birth [40]. Marginal and velamentous insertions are also associated with preterm birth in singletons [41]. In our oocyte donation group, VUE was more frequent, and although most pregnancies reached 34–35 weeks, earlier delivery might have occurred in the absence of clinical intervention.

Neonatal outcomes did not differ between groups, although oocyte donation has been associated with lower birthweight in twins [8]. Placental weight, size, and thickness showed no group differences, consistent with previous studies [21,23]. Although lower placental weight and surface area are linked to fetal growth restriction [42], this was not observed here, suggesting a multifactorial etiology. Umbilical cord characteristics (length, thickness, coiling, insertion, vessel number, true knots) did not differ significantly between groups, consistent with the literature [21,23]. Approximately 50% of cords had abnormal insertion (marginal or velamentous), a feature associated with ART [43]. In triplets, velamentous insertion has been linked to small-for-gestational-age neonates [44] and possibly to a lower risk of lethal vascular anastomoses [45]. Intraparenchymal infarcts and hematomas were more frequent in the oocyte donation group, as previously reported [19]. While Esteves et al. reported more placenta accreta in oocyte donation pregnancies [21], this was not observed in our series.

No group differences were observed in acute inflammatory lesions (chorioamnionitis, funisitis), consistent with other studies [21]. This supports the idea that oocyte donation is associated with chronic, rather than acute, immunological processes. VUE was the most frequent chronic inflammatory finding (8.2%) and significantly more common in the oocyte donation group, in line with prior studies [18,19]. While VUE is found in up to 10% of term placentas, our median gestational age was 33 weeks. Chronic deciduitis was also more common in the oocyte donation group, as reported previously [19,23], though not all studies have confirmed this [21]. An immunological mechanism likely underlies these lesions. Gundogan et al. found increased CD4+ T lymphocytes and CD56+ NK cells in the basal plates of oocyte donation placentas [23].

Accelerated villous maturation was the most frequent maternal malperfusion lesion (83.9%), with no group differences. Distal villous hypoplasia also did not differ significantly. Most studies report no differences in maternal malperfusion [18,19,23], although Esteves et al. found increased rates in oocyte donation cases [21]. Despite this, preeclampsia and fetal growth restriction—typically linked to this pattern—were more frequent in the oocyte donation group. Decidual arteriopathy did not differ between groups. Fetal malperfusion features were significantly more common in oocyte donation placentas, as previously reported [21]. Diminished ovarian reserve, common in this population, has also been linked to fetal malperfusion [39]. These lesions have been associated with fetal hypercoagulability and umbilical cord obstruction [46], potentially contributing to the higher rates of fetal growth restriction and fetal death observed.

Chorangiosis rates were similar across groups and represented the second most common lesion overall (66.1%), aligning with its known association with multiple pregnancies [47]. Chorangiomas were more frequent in the oocyte donation group, although this difference lost significance after adjustment. These lesions have been linked to higher maternal age and nulliparity [48], but these factors did not differ in our cohort. Other studies also reported no group differences in chorangioma or chorangiosis [21]. Villous edema, dystrophic calcifications, and nucleated red blood cells showed no significant differences, whereas intervillous fibrin deposition was more frequent in the oocyte donation group, consistent with its association with fetal growth restriction and death [25]. Prior studies have reported increased intervillous and basal plate fibrin in oocyte donation placentas [23], although others did not confirm this [21].

Strengths of our study include the relatively large sample size, thorough data collection, expert pathological review, and use of multivariate analyses. Limitations include its retrospective design and related biases. We lacked data on potential confounders such as maternal smoking, paternal age, and oocyte donor characteristics. Aspirin, known to reduce vascular complications [49], was only administered to oocyte donation patients from 2012 onward; data on its use in the control group were unavailable. Fresh and frozen embryos were analyzed together, though some studies suggest differences between them [50,51], while others do not [52]. Fresh and frozen embryos and all chorionicity types were analyzed together, given the limited number of cases, despite known differences in outcomes [32,50,51,52]. ART practices and perinatal care have evolved over the 20-year study period, though one study found no significant improvement in triplet outcomes [53]. More recent evidence from our own series suggests that baseline characteristics and obstetric management protocols have indeed changed over time, potentially influencing pregnancy outcomes [54]. Selection bias is possible, as only cases with placental evaluation were included, potentially omitting uneventful pregnancies. Missing placentas mainly corresponded to uncomplicated cases in which pathological examination was not requested, and therefore the analyzed cohort may overrepresent pregnancies with adverse outcomes. Placentas from pregnancies with both intrauterine death and surviving fetuses were analyzed together, though the former may have presented more abnormalities. Lastly, although some variables were missing, the overall proportion of missing data was low, and their exclusion from the corresponding analyses is unlikely to have materially affected the results. Macroscopic data were limited in older cases, particularly regarding vascular anastomoses. Full umbilical cord length was often unavailable, as obstetricians routinely ligate the cord at variable distances from the newborn. Therefore, only the placental segment could be measured in most cases, which represents a minor limitation when comparing cord lengths across groups.

## 5. Conclusions

Oocyte donation in triplet pregnancies is associated with a higher rate of complications and placental abnormalities when compared with ART without oocyte donation, reflecting complex and multifactorial mechanisms. Systematic placental examination is recommended in these cases to improve understanding and management. Further studies exploring immune and vascular pathways may support better clinical protocols and donor selection strategies.

## Figures and Tables

**Table 1:** Description of maternal and obstetric variables, statistical significance and factors associated with oocyte donation and non-oocyte donation triplet pregnancies.

Variable	Total, *n* = 77	Oocyte Donation, *n* = 29	Non-Oocyte Donation, *n* = 48	Signification (*p*)	Odds Ratio (OR)	IC 95%	Adjusted *p*	Adjusted OR	IC 95%
**Chorionicity**									
- MCTA	3 (3.9)	1 (3.4)	2 (4.2)	1.00 ^c^	0.82	0.07–9.48			
- DCTA	37 (48.1)	15 (51.7)	22 (45.8)	0.61 ^b^	1.26	0.50–3.18			
- TCTA	37 (48.1)	13 (44.8)	24 (50.0)	0.66 ^b^	0.81	0.32–2.04			
Presence of a MC component	40 (51.9)	16 (55.2)	24 (51.9)	0.66 ^b^	1.23	0.48–3.10			
**Maternal age (years)**	35.5 ± 4.1	34.7 ± 4.6	35.8 ± 3.6	0.53 ^a^	-	-			
**Pre-pregnancy BMI (kg/m^2^)**	24.2 ± 2.2	24.4 ± 2.0	24.1 ± 2.3	0.27 ^a^	-	-			
- BMI ≥ 30	1 (1.3)	0 (0)	1 (3.4)	0.20 ^c^	-	-			
**Year of conception**									
- 2000–2011	23 (29.9)	7 (24.1)	16 (33.3)	0.39 ^b^	1.57	0.55–4.45			
- 2012–2024	54 (70.1)	22 (75.9)	32 (66.7)						
**Parity**				0.06 ^c^	0.32	0.09–1.07			
- First gestation	57 (74.0)	25 (86.2)	32 (66.7)						
- Second or more gestation	20 (26.0)	4 (13.8)	16 (33.3)						
**Previous failed pregnancy attempts by ART**	28 (47.5)	16 (64.0)	12 (42.9)	**0.02 ^b^**	3.25	1.10–9-57	**0.03**	2.34	1.06–8.76

MCTA: monochorionic triamniotic; DCTA: dichorionic triamniotic; TCTA: trichorionic triamniotic; MC: monochorionic; BMI: body mass index; ART: assisted reproductive technology. ^a^ Data compared by Student’s *t* test. ^b^ Data compared by chi-squared test. ^c^ Data compared by Fisher’s exact test. Bold indicates statistical significant values.

**Table 2:** Description of maternal, fetal, obstetric and neonatal variables, statistical significance and factors associated with oocyte donation and non-oocyte donation triplet pregnancies.

Variable	Total, *n* = 77	Oocyte Donation, *n* = 29	Non-Oocyte Donation, *n* = 48	Signification (*p*)	Odds Ratio (OR)	IC 95%	Adjusted *p*	Adjusted OR	IC 95%
**Maternal complications**									
Preeclampsia	12 (15.6)	8 (27.6)	4 (8.3)	**0.02 ^b^**	4.19	1.13–15.49	**0.03**	5.02	2.24–13.42
Pregnancy-induced hypertension	8 (10.4)	6 (20.7)	2 (4.2)	**0.02 ^c^**	6.00	1.12–32.08	**0.03**	6.32	1.76–26.54
Gestational diabetes	8 (10.4)	3 (10.3)	5 (10.4)	0.99 ^c^	0.99	0.21–4.50			
Intrahepatic cholestasis	6 (7.8)	5 (17.2)	1 (2.1)	**0.01 ^c^**	9.79	1.08–88.60	0.12	10.41	2.13–67.65
Pregnancy-induced hypothyroidism	6 (7.8)	2 (6.9)	4 (8.3)	0.82 ^c^	0.81	0.14–4.75			
Iron deficiency anaemia	15 (19.5)	6 (20.7)	9 (18.8)	0.83 ^b^	1.13	0.35–3.58			
**Fetal complications**									
Twin-to-twin transfusion syndrome	3 (4.1)	2 (7.1)	1 (2.2)	0.29 ^c^	3.46	0.29–40.05			
Twin anemia-polycythemia sequence	0 (0.0)	0 (0.0)	0 (0.0)	**-**	**-**	**-**	**-**		
Amniotic fluid discordance	7 (9.5)	2 (7.1)	5 (10.9)	0.59 ^c^	0.63	0.11–3.49			
Biometry discordance	7 (9.5)	4 (14.3)	3 (6.5)	0.26 ^c^	2.38	0.49–11.57			
**Variable**	**Total, *n* = 231**	**Oocyte donation, *n* = 77**	**Non-oocyte donation, *n* = 136**	**Signification (*p*)**	**Odds Ratio (OR)**	**IC 95%**	**Adjusted *p***	**Adjusted OR**	**IC 95%**
Fetal growth restriction	26 (11.3)	15 (17.2)	11 (7.6)	**0.02 ^b^**	2.51	1.09–5.77	**0.04**	3.32	1.21–6.54
Fetal malformations	3 (1.4)	2 (2.4)	1 (0.7)	0.30 ^c^	3.34	0.29–37.42			
Fetal death	24 (10.4)	18 (20.7)	6 (4.2)	**<0.01 ^b^**	6.00	2.27–15.79	**0.01**	7.21	2.21–16.54
**Variable**	**Total, *n* = 77**	**Oocyte donation, *n* = 29**	**Non-oocyte donation, *n* = 48**	**Signification (*p*)**	**Odds Ratio (OR)**	**IC 95%**	**Adjusted *p***	**Adjusted OR**	**IC 95%**
**Obstetric complications**									
Threatened preterm labor	17 (23.0)	5 (17.9)	12 (26.1)	0.41 ^b^	0.61	0.19–1.98			
Premature rupture of membranes	13 (17.6)	4 (14.3)	9 (19.6)	0.56 ^c^	0.68	0.19–2.47			
**Variable**	**Total, *n* = 73**	**Oocyte donation, *n* = 26**	**Non-oocyte donation, *n* = 47**	**Signification (*p*)**	**Odds Ratio (OR)**	**IC 95%**	**Adjusted *p***	**Adjusted OR**	**IC 95%**
**Preterm birth**	33.0 (P25 = 31.0, P75 = 34.0)	32.5 (P25 = 30.0, P75 = 34.7)	33.0 (P25 = 31.0, P75 = 34.0)	0.91 ^a^	-	-			
Preterm birth (<34 weeks)	45 (61.6)	17 (60.7)	28 (62.2)	0.89 ^b^	0.93	0.35–2.47			
- <31.6 weeks	31 (42.5)	13 (46.4)	18 (40.0)	0.58 ^b^	1.30	0.50–3.37			
- Between 32.0–35.6 weeks	41 (56.2)	15 (53.6)	26 (57.8)	0.72 ^b^	0.84	0.32–2.17			
- >36.0 weeks	2 (2.7)	1 (1.4)	1 (2.2)	0.73 ^c^	1.63	0.09–27.14			
**Variable**	**Total, *n* = 213**	**Oocyte donation, *n* = 77**	**Non-oocyte donation, *n* = 136**	**Signification (*p*)**	**Odds Ratio (OR)**	**IC 95%**	**Adjusted *p***	**Adjusted OR**	**IC 95%**
**Neonatal complications**									
Birth weight (g)	1755 (P25 = 1545, P75 = 1965)	1670 (P25 = 1526, P75 = 2070)	1763 (P25 = 1550, P75 = 1943)	0.52 ^a^	-	-			
- <1500g	40 (21.1)	16 (22.5)	24 (20.2)	0.69 ^a^	1.15	0.56–2.35			
- 1500–2500g	144 (75.8)	52 (73.2)	92 (77.3)	0.52 ^a^	0.80	0.40–1.58			
- >2500g	6 (3.2)	3 (4.2)	3 (2.5)	0.34 ^a^	1.06	0.48–2.03			
Umbilical cord pH	7.33 (P25 = 7.30, P75 = 7.35)	7.34 (P25 = 7.31, P75 = 7.36)	7.32 (P25 = 7.29, P75 = 7.35)	0.06 ^a^	-	-			
- <7.20	11 (5.8)	2 (2.7)	9 (7.9)	0.13 ^c^	0.32	0.06–1.52			
Apgar score 5 min ≤5	13 (7.1)	6 (9.4)	7 (5.9)	0.38 ^b^	1.64	0.52–5.10			

^a^ Data compared by Mann–Whitney U test. ^b^ Data compared by chi-squared test. ^c^ Data compared by Fisher’s exact test. Bold indicates statistical significant values.

**Table 3:** Description of gross placental variables, statistical significance and factors associated with oocyte donation and non-oocyte donation triplet pregnancies.

Variable	Total, *n* = 77	Oocyte Donation, *n* = 29	Non-Oocyte Donation, *n* = 48	Signification (*p*)	Odds Ratio (OR)	95% IC	Adjusted *p*	Adjusted OR	95% IC
**Placental weight (g)**	859.74 ± 252.67	828.30 ± 339.45	878.28 ± 186.62	0.45 ^b^	-	**-**			
**Placental weight**									
- ≤10	13 (17.8)	7 (25.9)	6 (13.0)	0.16 ^c^	2.33	0.69–7.86			
- 11–50	23 (31.5)	6 (22.2)	17 (37.0)	0.19 ^c^	0.48	0.16–1.44			
- 51–89	18 (24.7)	8 (29.6)	10 (21.7)	0.45 ^c^	1.51	0.51–4.47			
- ≥90	19 (26.0)	6 (22.2)	13 (28.3)	0.57 ^c^	0.72	0.23–2.20			
**Placental size (cm)**	21.0 (P25 = 17.5, P75 = 28.5)	21.0 (P25 = 18.0, P75 = 29.0)	21.0 (P25 = 17.0, P75 = 30.0)	0.41 ^a^	**-**	**-**			
**Placental thickness (cm)**	2.1 (P25 = 2.0, P75 = 2.4)	2.1 (P25 = 2.0, P75 = 2.5)	2.2 (P25 = 2.0, P75 = 2.3)	0.65 ^a^	**-**	**-**			
**Variable**	**Total, *n* = 40**	**Oocyte donation, *n* = 16**	**Non-oocyte donation, *n* = 24**	**Signification (*p*)**	**Odds Ratio (OR)**	**CI 95%**	**Adjusted *p***	**Adjusted OR**	**95% IC**
**Superficial vascular anastomoses**	9 (42.9)	3 (50.0)	6 (40.0)	0.67 ^d^	1.50	0.22–10.97			
**Variable**	**Total, *n* = 231**	**Oocyte donation, *n* = 77**	**Non-oocyte donation, *n* = 136**	**Signification (*p*)**	**Odds Ratio (OR)**	**CI 95%**	**Adjusted *p***	**Adjusted OR**	**95% IC**
**Umbilicial cord insertion**									
- Central	17 (7.9)	3 (3.6)	14 (10.6)	0.06 **^d^**	0.31	0.08–1.13			
- Paracentral	82 (38.0)	35 (41.7)	47 (35.6)	0.37 ^c^	1.29	0.73–2.26			
- Marginal	68 (31.5)	26 (31.0)	42 (31.8)	0.84 ^c^	0.96	0.53–1.73			
- Velamentous	49 (22.7)	20 (23.8)	29 (22.0)	0.75 ^c^	1.11	0.58–2.12			
**Umbilical cord lenght**	18.0 (P25 = 13.0, P75 = 22.0)	18.0 (P25 = 13.0, P75 = 22.0)	18.0 (P25 = 12.0, P75 = 23.0)	0.97 ^a^	-	-			
**Umbilical cord thickness**	1.2 (P25 = 1.0, P75 = 1.4)	1.2 (P25 = 1.0, P75 = 1.4)	1.2 (P25 = 1.0, P75 = 1.47)	0.23 ^a^	-	-			
**Umbilical cord coiling**									
- Hypocoiling (<1)	9 (6.2)	5 (9.6)	4 (4.3)	0.20 ^d^	2.36	0.60–9.23			
- Normal (1–3)	121 (83.4)	44 (84.6)	77 (82.8)	0.77 ^c^	1.14	0.45–2.88			
- Hypercoiling (>3)	15 (10.3)	3 (5.8)	12 (12.9)	0.23 ^d^	0.41	0.11–1.53			
**Single umbilical artery**	10 (5.8)	4 (6.3)	6 (5.6)	0.86 ^d^	1.12	0.30–4.13			
**True umbilical knots**	4 (2.2)	0 (0.0)	4 (3.4)	0.12 ^d^	0.63	0.56–1.02			
**Variable**	**Total, *n* = 188**	**Oocyte donation, *n* = 70**	**Non-oocyte donation, *n* = 118**	**Signification (*p*)**	**Odds Ratio (OR)**	**CI 95%**	**Adjusted *p***	**Adjusted OR**	**95% IC**
**Membranous alterations**	5 (3.2)	2 (3.5)	3 (3.1)	0.88 ^d^	1.13	0.18–7.03			
**Variable**	**Total, *n* = 231**	**Oocyte donation, *n* = 77**	**Non-oocyte donation, *n* = 136**	**Signification (*p*)**	**Odds Ratio (OR)**	**CI 95%**	**Adjusted *p***	**Adjusted OR**	**95% IC**
**Intraparenchymatous infarct/hematoma**	36 (16.5)	20 (23.5)	16 (12.0)	**0.02 ^c^**	2.25	1.09-4.64	**0.04**	3.43	1.65–7.54
**Retroplacental hematoma**	0 (0.0)	0 (0.0)	0 (0.0)	**-**	-	-			
**Subchorionic hematoma**	5 (2.5)	2 (2.6)	3 (2.5)	0.94 ^d^	1.06	0.17-6.51			

^a^ Data compared by Mann–Whitney U test. ^b^ Data compared by Student’s *t* test. ^c^ Data compared by chi-squared test. ^d^ Data compared by Fisher’s exact test. Bold indicates statistical significant values.

**Table 4:** Description of histopathological placental variables, statistical significance and factors associated with oocyte donation and non-oocyte donation triplet pregnancies.

Variable	Total, *n* = 231	Oocyte Donation, *n* = 77	Non-Oocyte Donation, *n* = 136	Signification (*p*)	Odds Ratio (OR)	IC 95%	Adjusted *p*	Adjusted OR	IC 95%
**Inflammatory pattern findings**									
Acute chorioamnionitis	21 (9.9)	7 (9.3)	14 (10.1)	0.85 ^a^	0.91	0.35–2.36			
Acute funisitis	9 (5.2)	4 (7.0)	5 (4.3)	0.44 ^b^	1.69	0.43–6.55			
Chronic deciduitis	4 (2.3)	4 (6.7)	0 (0)	**0.01 ^b^**	2.33	0.27–0.41	**0.03**	3.24	1.32–8.78
Chronic villitis (UEV)	15 (8.2)	10 (15.2)	5 (4.3)	**0.02 ^b^**	4.00	1.30–12.26	**0.02**	4.21	1.56–10.65
Chronic intervillitis	3 (1.7)	3 (5.0)	0 (0)	**0.03 ^b^**	0.32	0.26–0.40	0.06	0.45	0.34–4.34
**Vascular pattern findings** Maternal vascular malperfusion									
Accelerated villous maturation	146 (83.9)	50 (82.0)	96 (85.0)	0.69 ^a^	0.80	0.35–1.84			
Distal villous hypoplasia	26 (14.9)	9 (14.8)	17 (15.0)	0.95 ^a^	0.97	0.40–2.34			
Decidual arteriopathy	6 (3.4)	3 (4.8)	3 (2.7)	0.46 ^b^	1.83	0.35–9.36			
Fetal vascular malperfusion									
Vascular trombi	7 (3.9)	5 (8.1)	2 (1.7)	**0.03 ^b^**	5.13	0.96–27.26	0.08	6.54	2.41–15.57
Avascular villi	11 (5.9)	9 (13.6)	2 (1.7)	**<0.01 ^b^**	9.23	1.93–44.15	**0.02**	7.82	2.21–19.09
Stem vessels obliteration	7 (3.9)	5 (8.1)	2 (1.7)	**0.03 ^b^**	5.13	0.96–27.26	0.06	4.42	0.21–13.23
Stromal-vascular karyorrhexis	11 (5.9)	9 (13.6)	2 (1.7)	**<0.01 ^b^**	9.23	1.93–44.15	**0.01**	8.65	3.01–18.76
Intramural fibrin deposits	3 (1.3)	3 (3.6)	0 (0)	**0.02 ^b^**	0.36	0.30–0.42	0.10	0.42	0.08–1.87
**Other findings**									
Villous edema	46 (26.9)	12 (21.1)	34 (29.8)	0.22 ^a^	0.62	0.29–1.33			
Chorangiosis	115 (66.1)	35 (61.4)	80 (68.4)	0.36 ^a^	0.73	0.38–1.42			
Chorangioma	11 (6.4)	7 (11.9)	4 (3.5)	**0.03 ^b^**	3.70	1.03–13.20	0.06	4.56	0.96–10.45
Dystrophic calcifications	40 (29.2)	9 (20.5)	3 (33.3)	0.12 ^b^	0.51	0.22–1.20			
Intervillous fibrin deposits	84 (38.9)	37 (50.0)	64 (33.1)	**0.01 ^b^**	2.02	1.13–3.59	**0.02**	2.78	1.21–6.56
Nucleated red blood cells	9 (5.0)	3 (5.0)	6 (5.0)	1.00 ^b^	1.00	0.24–4.14			

^a^ Data compared by chi-squared test. ^b^ Data compared by Fisher’s exact test. UEV: unknown etiology villitis. Bold indicates statistical significant values.

## Data Availability

The datasets generated and/or analysed during the current study are available from the corresponding author on reasonable request.
